# Test-Retest Reliability of an Automated Infrared-Assisted Trunk Accelerometer-Based Gait Analysis System

**DOI:** 10.3390/s16081156

**Published:** 2016-07-23

**Authors:** Chia-Yu Hsu, Yuh-Show Tsai, Cheng-Shiang Yau, Hung-Hai Shie, Chu-Ming Wu

**Affiliations:** 1 Department of Rehabilitation Medicine, Ten-Chan General Hospital, No. 155, Yanping Rd., Zhongli Dist., Taoyuan City 320, Taiwan; f927kimo@gmail.com (C.-Y.H.); drwjm@yahoo.com.tw (C.-M.W.); 2 Department of Biomedical Engineering, Chung Yuan Christian University, No. 200, Zhongbei Rd., Zhongli Dist., Taoyuan City 320, Taiwan; specially527@gmail.com; 3 Department of Physiotherapy, Ten-Chan General Hospital, No. 155, Yanping Rd., Zhongli Dist., Taoyuan City 320, Taiwan; milla1220@yahoo.com.tw

**Keywords:** accelerometry, gait, infrared, trunk acceleration, reliability

## Abstract

The aim of this study was to determine the test-retest reliability of an automated infrared-assisted, trunk accelerometer-based gait analysis system for measuring gait parameters of healthy subjects in a hospital. Thirty-five participants (28 of them females; age range, 23–79 years) performed a 5-m walk twice using an accelerometer-based gait analysis system with infrared assist. Measurements of spatiotemporal gait parameters (walking speed, step length, and cadence) and trunk control (gait symmetry, gait regularity, acceleration root mean square (RMS), and acceleration root mean square ratio (RMSR)) were recorded in two separate walking tests conducted 1 week apart. Relative and absolute test-retest reliability was determined by calculating the intra-class correlation coefficient (ICC_3,1_) and smallest detectable difference (SDD), respectively. The test-retest reliability was excellent for walking speed (ICC = 0.87, 95% confidence interval = 0.74–0.93, SDD = 13.4%), step length (ICC = 0.81, 95% confidence interval = 0.63–0.91, SDD = 12.2%), cadence (ICC = 0.81, 95% confidence interval = 0.63–0.91, SDD = 10.8%), and trunk control (step and stride regularity in anterior-posterior direction, acceleration RMS and acceleration RMSR in medial-lateral direction, and acceleration RMS and stride regularity in vertical direction). An automated infrared-assisted, trunk accelerometer-based gait analysis system is a reliable tool for measuring gait parameters in the hospital environment.

## 1. Introduction

The overall prevalence of gait disorder in a population-based cohort of 488 older (ages 60 to 97 years) community-dwelling participants was 32% [1]. Studies have shown that 60% of hospitalized patients with neurologic disorders and 50% of those in a nursing home had a gait disturbance [2,3]. Patients with gait disorders suffer from reduced mobility, diminished quality of life, falls, major fractures, head trauma, and reduced survival [4,5]. As many as 30% of people aged 65 years and older fall each year [6]. In a pooled analysis of nine cohort studies, gait speed was associated with survival, with a hazard ratio per 0.1 m/s gait speed of 0.88 (95% CI 0.87–0.90) [7]. Therefore, clinical gait analysis is crucial in determining the diagnosis, severity, progress, and prognosis of patients with gait disorder [8].

In routine clinical practice, establishing the reliability of gait analysis is essential for clinicians to evaluate and interpret the measurement results [8]. Clinical gait measurements should be reproducible, stable, accurate, capable of distinguishing between normal and abnormal conditions, and cost-effective [8]. In current clinical practice, most clinicians rely largely on descriptive terms to evaluate gait, such as hemiparetic, steppage, or ataxic gait, but do not use quantitative measures. Furthermore, conventional lab-based equipment techniques, such as 3-dimensional motion capturing systems or force plates, though considered the gold standard for gait analysis, were restricted in their clinical application due to tedious data acquisition, costly lab equipment, and the need for specially trained personnel [9,10]. In contrast, accelerometers have a potential role in clinical gait analysis because they are wearable, wireless, and non-obtrusive [11]. It takes only 10 min to perform an accelerometer-based gait analysis (AGA) in a normal corridor or walkway [12]. Several clinically relevant gait parameters, such as cadence, step count, gait symmetry, and gait regularity can also simultaneously be derived from acceleration signals using autocorrelations or peak detection algorithms [13,14]. In addition, accelerometer-based gait analysis is validated in previous studies with ground reaction force measurements by treadmill [14,15] and force plate [16].

In the current literature, few studies have investigated the test-retest reliability of the AGA system. Henriksen et al. reported excellent intra-class correlation coefficient (ICC) values (0.77–0.96) for accelerometer-based measurement of basic spatiotemporal gait parameters (i.e., cadence, stride length, step length, and acceleration root mean square (RMS) value) [9]. A 10-m walking distance of an indoor hospital was arranged with assistance of photoelectric sensors. Senden et al. reported excellent repeatability for acceleration-based measurement of basic gait parameters (i.e., step length, cadence, speed, step time) (ICC = 0.902–0.997) but lower repeatability for gait symmetry and regularity (ICC = 0.509–0.787) [12]. Their participants walked a 20-m distance in a hospital without infrared (IR) assist. Maffiuletti et al. found excellent test-retest reliability for walking speed, cadence, step length, and stride length (ICC = 0.988–0.994) in a laboratory [17]. Although these studies had demonstrated excellent reliability of the accelerometer-based system for basic spatiotemporal gait parameters and acceleration RMS, no studies reported the reliability of gait symmetry, gait regularity with autocorrelation methods [13], and acceleration root mean square ratio (RMSR), or the degree of body sway [11]. Moreover, the limited and crowded space in a hospital environment may disturb clinical gait analysis. Therefore, developing a reliable and relatively short-distance accelerometer-based system is crucial for clinical gait analysis. We hypothesized that the gait parameters derived from the accelerometer-based system are reliable in the hospital and they are much better with IR assist than without in a total 8-m walk.

The major aim of this study was to investigate the test-retest reliability of an automated infrared-assisted, accelerometer-based gait analysis system of healthy participants in a clinical setting. The minor aim was to compare these findings to the results obtained without infrared assist.

## 2. Materials and Methods

### 2.1. Participants

Healthy adults were recruited from the rehabilitation department of a teaching hospital in northern Taiwan. Inclusion criteria were the following: (1) age ≥ 20 years; (2) the ability to accept and follow verbal instructions; and (3) the ability to walk independently without walking aids. Exclusion criteria were the following: (1) any systemic disease (e.g., diabetes, hypertension, or rheumatic disease); (2) any neurologic disease that may affect gait (e.g., Parkinsonism, stroke, or ataxia); (3) prior spine or lower extremities surgery; and (4) any comorbid condition that interferes with gait. All participants agreed to participate in the study and signed an informed consent form prior to examination. The study was approved by the medical ethics committee of the hospital.

### 2.2. Experimental Protocol

**Figure 1:**
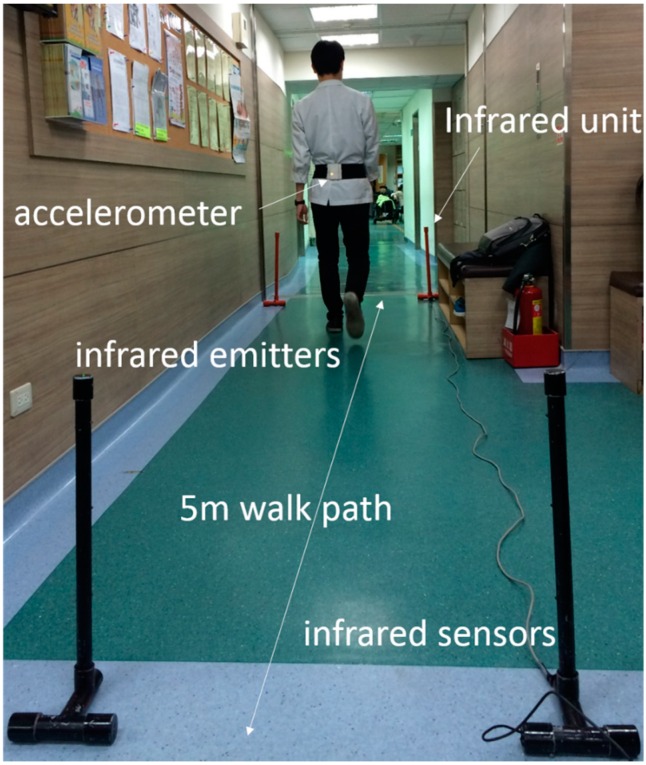
The wireless accelerometer unit was attached to the L3–L4 spinal segment of the lower back using an elastic belt.

**Figure 2:**
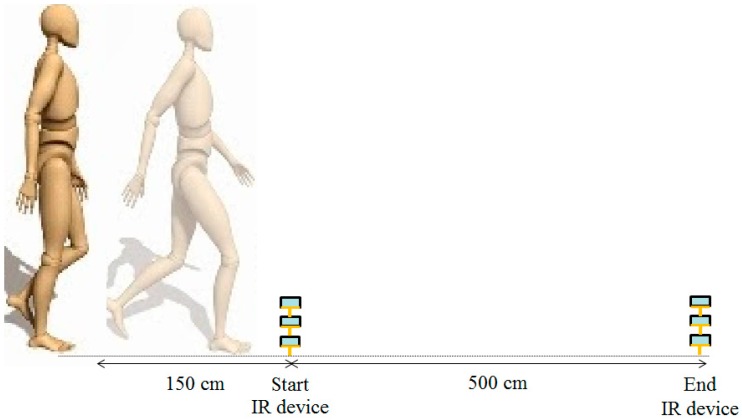
Infrared (IR) devices are placed at each end of a 5-m walk path to concurrently collect trunk accelerometric data when a participant passes the IR units between the start line and the end line. Participants are allowed 1.5 m for gait initiation and termination.

To determine test-retest reliability, study participants were tested with an interval of 1 week in between tests. Both assessments were performed at the same time of the day, at the same location, and by the same assessor. All participants walked twice at a self-selected speed along the corridor on a 5-m walking path with an even surface in the hospital’s department of rehabilitation (Figure 1). The participants wore their usual shoes, excluding sandals, slippers, and high-heeled shoes. The wireless accelerometer unit was attached to the L3–L4 spinal segment of the lower back using an elastic belt. This position was chosen owing to its proximity to the center of mass, provided that the sensor is kept in the midline [13]. Participants were allowed 1.5 m for gait initiation and termination and 8 m for total walk. The middle 5 m was used for analysis and determined automatically by the system with and without IR assist (Figure 2). Participants were instructed to walk safely in a usual manner. The results of measurements were recorded and analyzed immediately by the system after each walk. The accelerometer was calibrated through the registor inside the tri-axial accelerometer ADXL345 before each test, and all values of tri-axial accelerometric data would become zero as a datum point, which was the benchmark for the following recorded accelerometric values. To test the system without IR assist, the walking time was manually recorded.

### 2.3. Equipment

**Figure 3:**
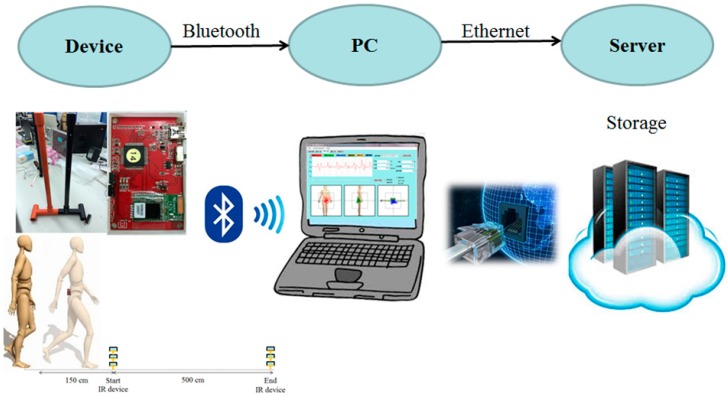
The IR-assist accelerometer-based gait analysis system has a wireless sensor unit, an IR unit, a laptop, and cloud storage database. PC, personal computer.

**Figure 4:**
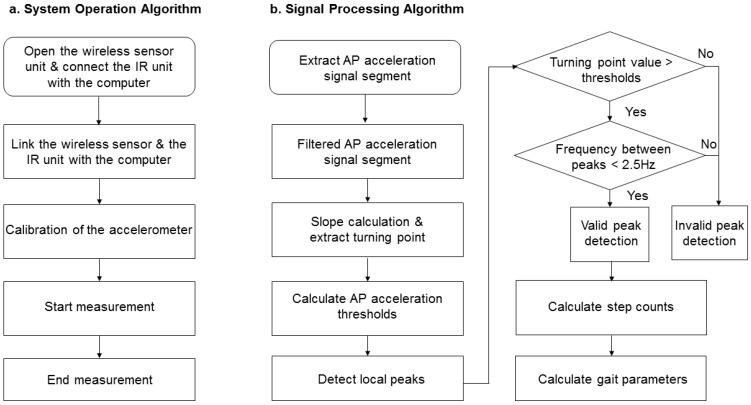
Flowchart of the system operation algorithm (**a**) and acceleration signal-processing algorithm (**b**). AP, anterior-posterior.

The IR-assisted, accelerometer-based gait analysis system is composed of a wireless sensor unit, an IR unit, a laptop, and a cloud storage database (Figure 3). The wireless sensor unit contains a tri-axial accelerometer (ADXL345, Analog Devices, Norwood, MA, USA), a micro control processor (MSP430F5529, Texas Instruments, Dallas, TX, USA), a Bluetooth module (BlueMode+B20, Stollmann, Hamburg, Germany), and a lithium battery. The tri-axial accelerometer can sense accelerometric changes in the anterior-posterior (AP), medial-lateral (ML), and vertical (V) directions, and its characteristics permit a highly sensitive measuring range of ±2 g. The size of the wireless sensor unit (length × width × height) measures 69.5 × 45.5 × 14.5 mm and the accelerometric data is digitized at a sample rate of 100 Hz. The battery capacity is 200 mAh and allows 6 h of continuous use. The IR unit has a micro-control processor Arduino nano with IR sensors (QJ-PT334-6B3, Tai-Ruey, Taichung, Taiwan) and IR emitters (QJ-IR333C-A, Tai-Ruey) placed at each end of the 5-m walk path at the midpoint, which allows the system to automatically record and analyze the accelerometric data (Figure 2). The communication and data transmission between the laptop and accelerometer is through Bluetooth via the UART interface of a MSP430F5529 micro-control processor, and between the laptop and IR unit by transmission line via the UART interface of an Arduino Nano 3.0 with ATmega328 micro-control processor (Atmel, San Jose, CA, USA). After calibration of the accelerometer, the system started recording the acceleration data on the laptop while receiving IR signals at the start line, and ended recording the data while receiving IR signals at the end line. All data is automatically and wireless transferred and stored in the laptop and a central server, and is used to produce a real-time gait analysis result (Figure 3). Flow charts of the system operation and signal-processing algorithm are illustrated (Figure 4).

### 2.4. Gait Characteristics

*Gait Speed* was calculated by dividing the 5-m walking distance by the walking time automatically measured by the IR units at each end of the 5-m walk path. Without IR assist, the walking time was recorded manually.

The *Number of steps* was determined by the following formulas:

(1) Total step count=Integer step count+The initial step+The last step

**Figure 5:**
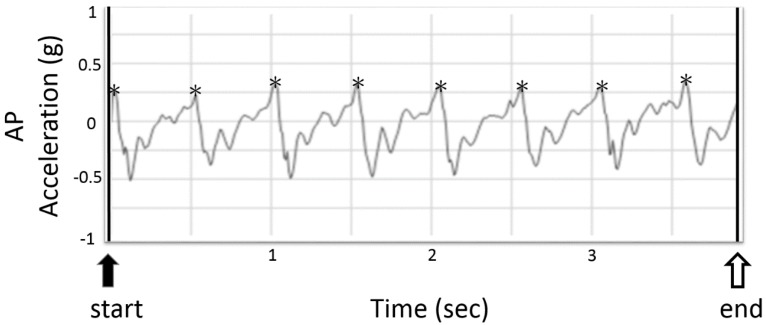
The typical plot of anterior-posterior (AP) acceleration signals from the tri-axial accelerometer during 5 m of normal walking samples at 100 Hz. The two black bars represent the moment of infrared-defined gait initiation and termination and the black arrowhead indicates the start of the measurement. The white arrowhead indicates the end of the measurement. The asterisks around peak acceleration values indicates initial foot contact of each integer step.

Integer step count was detected by AP peak acceleration detection algorithms [15,16]. The peak of the AP acceleration value was used as the point of initial foot contact (Figure 5). The first and the last step were probably not an integer step. The initial step was estimated with the time interval between the start line and the first integer step divided by the average time interval between each step. The last step was estimated with the time interval between the last integer step and the end line divided by the average time interval between each step. Total step counts were calculated to the tenths place value from the tri-axial acceleration data with IR assist. Only the integer step count could be calculated by the system.

*Cadence* was calculated by dividing the total step count by the walking time. 

*Step length* was calculated by dividing the 5-m distance divided by the total number of steps taken.

*Acceleration Root mean square (RMS)* in the AP, ML, and V directions was utilized to represent the average acceleration along each three-dimensional axis during the 5-m walking period.

*Acceleration Root mean square ratio (RMSR)* in the ML direction was calculated by dividing the RMS of the acceleration vector in the ML direction by the corresponding RMS acceleration vector magnitude [18]. The acceleration RMSR in the ML direction is associated with walking balance and has a common value at the preferred walking speed of healthy participants that can be used as a threshold for detecting gait abnormalities [11,18].

*Gait Regularity* of steps and stride in AP, ML, and V directions was estimated from the autocorrelation coefficient method proposed by Moe-Nilssen et al. [13]. The autocorrelation coefficient refers to the correlation of a time series with its own past or future values. The closeness of the autocorrelation coefficient to 1.0 reflects high step or stride regularity [19].

*Gait Symmetry* in AP, ML, and V directions was derived from the ratio of the stride regularity to the step regularity [13]. We expressed this ratio as a percentage, where closeness to 100% reflected high gait symmetry.

### 2.5. Statistical Analysis

Nonparametric Kolmogorov-Smirnov testing was utilized to verify the normality of the data distributions prior to parametric testing. Paired sample t-tests were used to determine systematic differences between separate test sessions. Relative reliability was expressed as two-way mixed single-measures type absolute intra-class correlation coefficients (ICC_3,1_) with associated 95% confidence intervals. Reliability or agreement was considered excellent above ICC values of 0.75, good between values of 0.59 and 0.75, fair between values of 0.40 and 0.58, and poor for values below 0.40 [16,20]. The smallest detectable difference (SDD) was calculated from the standard error of measurement (SEM) to quantify the absolute reliability of the measures [21,22]. SEM and SDD values and their corresponding 95% confidence intervals were calculated using the following formulas [23]:

(2) $$\text{SEM}=SD_{\text{all test scores}}\times \sqrt{1-r_{test-retest}}$$

(3) $$\text{SDD}=1.96\times \sqrt{2}\times SEM$$

where *SD* is the standard deviation of all test scores and *r*_test-retest_ is the ICC_3,1_ derived test-retest reliability. The SDD can also be expressed as a percentage of the SDD divided by mean of measurements [24]. We used Bland-Altman plots with 95% confidence interval agreement limits to visualize the agreement between two repeated measurements [25]. All data were analyzed with IBM SPSS 22.0 Statistics software.

## 3. Results

In total, 35 healthy adults (Age: 23–79 years, Height: 161.5 ± 7.9 cm, Weight: 61.6 ± 13.9 kg) consisting of 7 men and 28 women agreed to participate in the study. All adults were able to perform the test on two separate occasions in accordance with test requirements. The method of measuring total step count was validated by a video motion analysis and the error for total step count was 0.1 step. Characteristics of the study participants are shown (Table 1).

**Figure 6:**
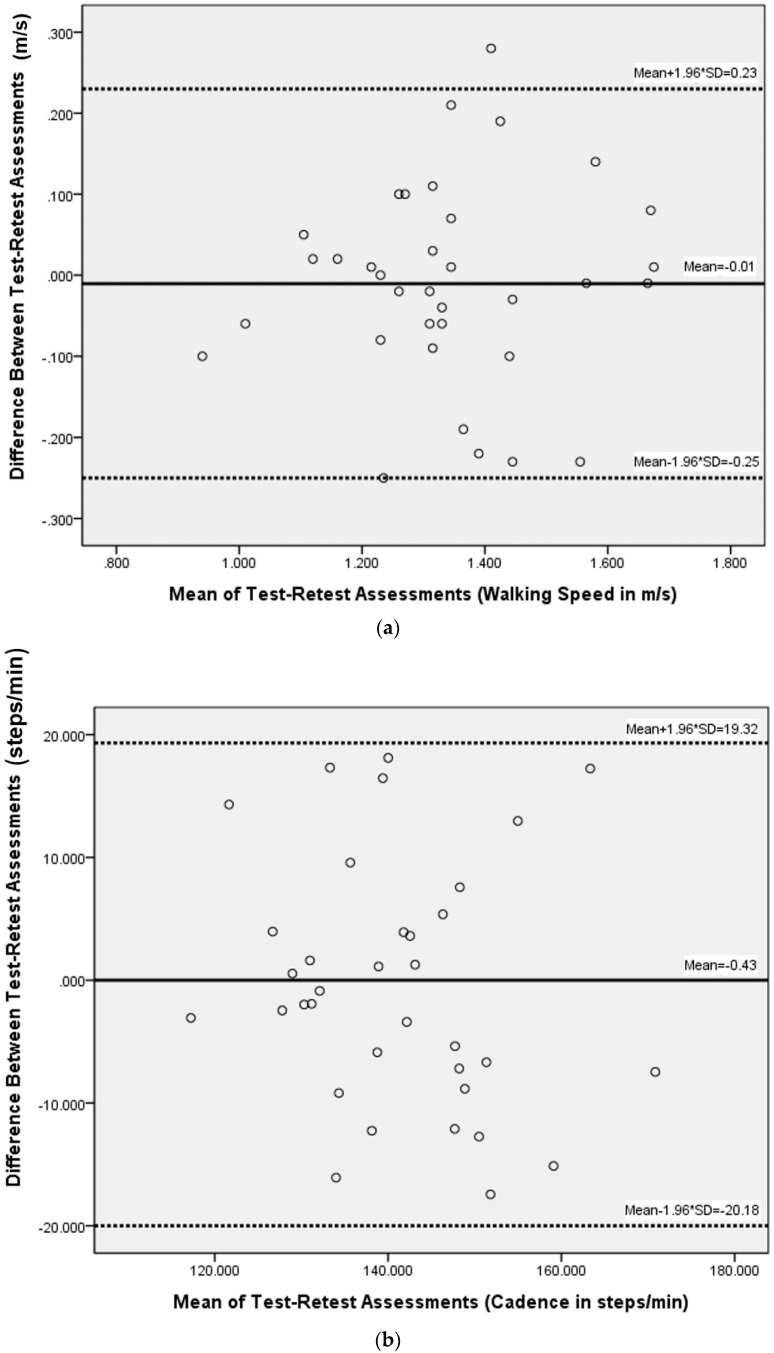

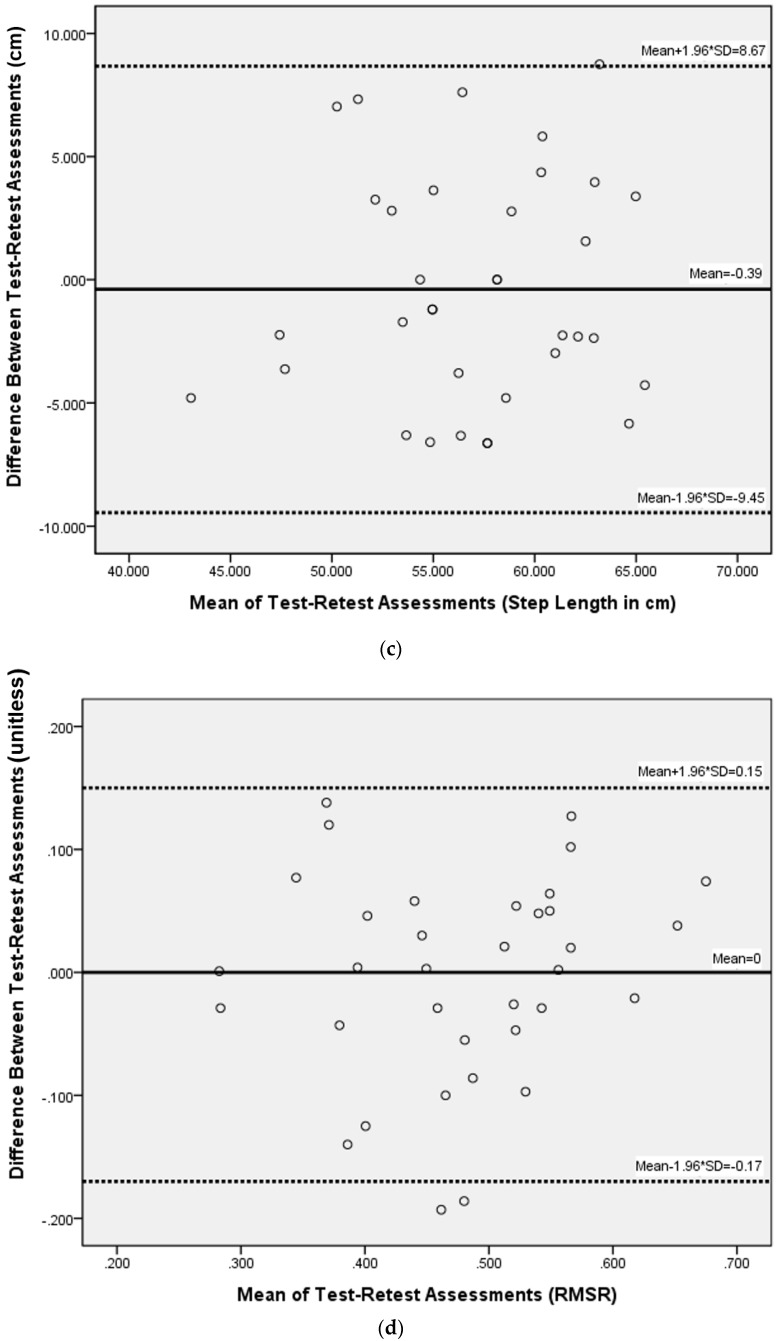
Bland-Altman method for plotting the differences in measures between the two walking tests against the corresponding mean of that measure for (**a**) walking speed; (**b**) cadence; (**c**) step length; and (**d**) acceleration RMSR in the medial-lateral direction. RMSR, root mean square ratio.

With IR assist, the test-retest reliability analysis including ICC_3,1_, SEM, and SDD values are shown (Table 2). Paired t-test showed no significant differences between the two tests for all measured parameters. ICC_3,1_ values were 0.87 (m/s) for velocity, 0.81 (step/min) for cadence, 0.81 (cm) for step length, and 0.81 (unitless) for the acceleration RMSR in the ML direction. ICC_3,1_ values for acceleration RMS were 0.74, 0.83, 0.88 in AP, ML, and V directions, respectively. SDD values were 0.18 (m/s) (13.4%) for velocity, 15.18 (steps/min) (10.8%) for cadence, 6.97 (cm/step) (12.2%) for step length, and 0.13 (unitless) (26%) for the acceleration RMSR in ML direction. SDD values for acceleration RMS were 0.061 (44.5%), 0.055 (41.1%), and 0.042 (21.4%) in AP, ML, and V directions, respectively. The Bland-Altman plots of basic spatiotemporal parameters (walking speed, cadence, step length) and acceleration RMSR showed that the data were not heteroscedastic (Figure 6). Limits of agreement were −0.25 and 0.23 (m/s) for walking velocity, −20.18 and 19.32 (step/min) for cadence, −9.45 and 8.67 (cm) for step length, and −0.17 and 0.15 (unitless) for the acceleration RMSR in the ML direction.

Values of ICC_3,1_ for step regularity were 0.79, 0.64, and 0.55; ICC_3,1_ values for stride regularity were 0.83, 0.72, and 0.76; and gait symmetry were 0.63, 0.38, 0.62, in the AP, ML, and vertical directions, respectively. The range of SDD values for gait symmetry, step regularity, and stride regularity were higher in the ML direction (SDD: 34.2%–43.2%) than in the AP or V directions (SDD: 14.7%–25.7%). Without IR assist, the test-retest reliability was excellent for walking speed (ICC = 0.77), but good for step length (ICC = 0.60) and poor for cadence (ICC = 0.35).

## 4. Discussion

This study showed that an automated IR-assisted, accelerometer-based gait analysis (AGA) system had excellent test-retest reliability in measuring spatiotemporal gait parameters (velocity, cadence, and step length) and trunk control (step and stride regularity in AP direction, acceleration RMS and acceleration RMSR in ML direction, and acceleration RMS and stride regularity in V direction) in a clinical setting. The reliability of gait regularity and symmetry measures was lower in the ML direction than the AP or vertical directions. Spatiotemporal gait parameters derived from the accelerometric data were much more reliable with IR assist than without IR use.

Few reliability studies on the AGA system of healthy participants had been performed in a hospital. Henriksen et al. arranged a total 10 m walk with photoelectric sensor assist in a hospital. Excellent ICC values (0.77–0.96) for cadence, stride length, step length, and acceleration root mean square (RMS) value were reported [9]. Senden et al. used a 20 m walk without IR assist in the hospital environment. Excellent repeatability for basic gait parameters (i.e., step length, cadence, speed, step time) (ICC = 0.902–0.997) but lower repeatability for gait symmetry and regularity (ICC = 0.509–0.787) were found [12]. They used the difference between the right and left step time divided by the bilateral average to estimate gait symmetry, and the standard deviations of the right and left step time to evaluate gait regularity, but not the autocorrelation method proposed by Moe-Nilssen et al. [13]. The excellent results of our studies were in accordance with previous AGA reliability studies regarding spatiotemporal gait parameters (velocity, cadence, step length) and trunk control (acceleration RMS). To our best knowledge, no accelerometric studies reported the reliability of gait symmetry and regularity with autocorrelation methods [13], the acceleration RMSR, and the degree of body sway [11] in clinical settings. Our study for the first time demonstrated that the IR-assist AGA system had excellent reliability of trunk control (step and stride regularity in AP direction; acceleration RMS and acceleration RMSR in ML direction; acceleration RMS and stride regularity in V direction). These findings suggested that the IR-assist, AGA system has its suitability for clinical gait analysis.

In our study, the absolute reliability for gait symmetry in the ML direction is considerably larger (SDD = 34.2%) than that in the AP (SDD = 18.8%) or V (SDD = 16.3%) direction, which suggests that caution should be taken when evaluating symmetry in the ML direction by the auto-correlation method. These findings are in accordance with a previous study presented by Tura et al. [26], which demonstrated that autocorrelation coefficients calculated in the AP and vertical directions showed superior sensitivity to those in the ML direction. The lower reliability for symmetry in the ML direction might be associated with natural functional differences between the lower extremities, such as laterality [27], pelvic rotation in the transverse plane [28], or smaller ground reaction force in the ML direction than the vertical direction during walking [29]. Therefore, in the ML direction, the acceleration RMSR may be a more appropriate measure than symmetry and regularity in accelerometer-based gait analysis. Conversely, gait symmetry and regularity in the AP and V directions are more reliable measures than in the ML direction in that they demonstrate relatively lower SDD values.

There are several advantages to combining IR sensors with an AGA system, especially when walking distance may be limited in the hospital environment. First, IR could make the accelerometer-base system more reliable than without IR assist. Our study showed spatiotemporal gait parameters derived from the accelerometric data were much more reliable with IR assist than without IR use. One can automatically detect walking time by IR and avoid the likelihood of measurement error associated with the stopwatch manual method, which depends largely on the examiner’s skill [30]. Second, IR can unambiguously define the moment of gait initiation and termination (Figure 4), which makes it possible to calculate step count accurately to one decimal point through the accelerometric data of the initial and the last step per measurement. The accuracy of the total step count is important, especially when a participant walks a short distance, because the accuracy of basic spatiotemporal gait parameters, such as cadence or step length, was largely dependent upon accurate step count measurement. Using the peak AP acceleration algorithms or the autocorrelation method alone could only estimate integer walking steps [13]. Third, the IR unit in our system was only 60 cm in height, making it portable and highly accessible when combining the AGA system.

Previous studies have shown the significance of the acceleration RMSR in the ML direction in detecting differences with accelerometer-based gait analysis [11,18]. Sekine et al. stated that comparing the acceleration RMSR in the ML direction as a measure of the degree of body sway between normal and hemiplegic participants revealed significant differences. The RMSR in the ML direction is representative of balance control, whereas the RMSR in the AP or vertical direction is associated with forward movement [18]. This difference renders the RMSR in the ML direction a more effective measure for detecting differences through gait analysis. Furthermore, Matsushima et al. found that the RMSR in the ML direction is significantly different in ataxic patients compared to healthy control participants [11]. Our study demonstrates that acceleration RMSR in the ML direction is a reliable indicator, measured by an IR-assisted, AGA system in a 5-m walk, which suggests its potential role in clinical rehabilitation.

There are some limitations to this study. The number of participants was small, albeit comparable with other reliability studies [9,12]. Although limited repositioning errors on the lumbar spine have been reported, there may have been variability in the positioning of the accelerometer with the elastic belt between the two assessments due to positioning errors [31].

## 5. Conclusions

An automated IR-assisted, accelerometer-based gait analysis system is a reliable tool for assessing spatiotemporal gait parameters and trunk control (step and stride regularity in AP direction, acceleration RMS and acceleration RMSR in ML direction, and acceleration RMS and stride regularity in V direction) of healthy participants in the hospital. Spatiotemporal gait parameters derived from the accelerometric data were much more reliable with IR assist than without IR use in a 5-m walk. Future reliability studies regarding clinical application of this system in different groups of patients are warranted.

## Figures and Tables

**Table 1:** Arthropometric data of the study participants.

	Mean (SD)	Min.	Max.
Age (years)	40.0 (15.2)	23	79
Height (cm)	161.5 (7.9)	150	183
Weight (kg)	61.6 (13.9)	43	95

**Table 2:** Results for reliability assessment of IR-assisted accelerometer-based system in measuring gait parameters in healthy participants.

		Occasion 1, Mean (SD)	Occasion 2, Mean (SD)	Paired *t* Test	ICC (95% Confidence Interval)	SEM	SDD	SDD, %
Velocity (m/s)		1.34 (0.19)	1.35 (0.18)	0.62	0.87 (0.74–0.93)	0.065	0.180	13.4
Step length (cm)		56.83 (5.95)	57.22 (5.67)	0.62	0.81 (0.63–0.91)	2.516	6.974	12.2
Cadence (step/min)		140.87 (11.85)	141.30 (13.40)	0.80	0.81 (0.63–0.91)	5.475	15.176	10.8
AP	acceleration RMS (g)	0.14 (0.41)	0.13 (0.43)	0.09	0.74 (0.48–0.87)	0.022	0.061	44.5
	Symmetry	81.20 (8.61)	83.30 (7.38)	0.15	0.63 (0.27–0.81)	4.884	13.538	16.5
	Stride regularity	0.58 (0.09)	0.60 (0.10)	0.32	0.83 (0.67–0.92)	0.040	0.111	18.8
	Step regularity	0.72 (0.08)	0.72 (0.09)	0.82	0.79 (0.59–0.89)	0.038	0.105	14.7
ML	acceleration RMS (g)	0.13 (0.05)	0.14 (0.05)	0.89	0.83 (0.66–0.91)	0.020	0.055	41.1
	acceleration RMSR	0.48 (0.11)	0.48 (0.10)	0.80	0.81 (0.62–0.90)	0.045	0.125	26.0
	Symmetry	81.46 (11.43)	79.49 (13.76)	0.46	0.38 (−0.23–0.69)	9.918	27.491	34.2
	Stride regularity	0.35 (0.09)	0.36 (0.09)	0.75	0.72 (0.45–0.86)	0.049	0.136	38.2
	Step regularity	0.44 (0.12)	0.46 (0.12)	0.30	0.64 (0.28–0.82)	0.070	0.194	43.2
V	acceleration RMS(g)	0.19 (0.04)	0.20 (0.05)	0.10	0.88 (0.76–0.94)	0.015	0.042	21.4
	Symmetry	84.24 (7.15)	81.76 (8.58)	0.09	0.62 (0.25–0.81)	4.894	13.565	16.3
	Stride regularity	0.56 (0.09)	0.55 (0.11)	0.36	0.76 (0.52–0.88)	0.049	0.136	24.4
	Step regularity	0.67 (0.08)	0.67 (0.11)	0.85	0.55 (0.11–0.77)	0.062	0.172	25.7

*IR* infrared, *ICC* interclass correlation coefficient; *SEM* standard error of measurement; *SDD* smallest detectable difference; *AP* anterior-posterior; *ML* medial-lateral; *V* vertical; *RMS* root mean square; *RMSR* root mean square ratio.

## Abbreviations

The following abbreviations are used in this manuscript:

AP	anterior-posterior
AGA	accelerometer-based gait analysis
ICC	intraclass correlation coefficient
IR	infrared
ML	medial-lateral
RMS	root mean square
RMSR	root mean square ratio
SDD	smallest detectable difference
SEM	standard error of measurement

## References

[1] Mahlknecht P., Kiechl S., Bloem B.R., Willeit J., Scherfler C., Gasperi A., Rungger G., Poewe W., Seppi K. (2013). Prevalence and burden of gait disorders in elderly men and women aged 60–97 years: A population-based study. PLoS ONE.

[2] Stolze H., Klebe S., Baecker C., Zechlin C., Friege L., Pohle S., Deuschl G. (2005). Prevalence of gait disorders in hospitalized neurological patients. Mov. Disord..

[3] Tinetti M.E., Baker D.I., McAvay G., Claus E.B., Garrett P., Gottschalk M., Koch M.L., Trainor K., Horwitz R.I. (1994). A multifactorial intervention to reduce the risk of falling among elderly people living in the community. N. Engl. J. Med..

[4] Snijders A.H., van de Warrenburg B.P., Giladi N., Bloem B.R. (2007). Neurological gait disorders in elderly people: Clinical approach and classification. Lancet Neurol..

[5] Sudarsky L. (2001). Gait disorders: Prevalence, morbidity, and etiology. Adv. Neurol..

[6] Nevitt M.C., Cummings S.R. (1993). Type of fall and risk of hip and wrist fractures: The study of osteoporotic fractures. The study of osteoporotic fractures research group. J. Am. Geriatr. Soc..

[7] Studenski S., Perera S., Patel K., Rosano C., Faulkner K., Inzitari M., Brach J., Chandler J., Cawthon P., Connor E.B. (2011). Gait speed and survival in older adults. JAMA.

[8] Baker R. (2006). Gait analysis methods in rehabilitation. J. Neuroeng. Rehabil..

[9] Henriksen M., Lund H., Moe-Nilssen R., Bliddal H., Danneskiod-Samsoe B. (2004). Test-retest reliability of trunk accelerometric gait analysis. Gait Posture.

[10] Howell D., Osternig L., Chou L.S. (2015). Monitoring recovery of gait balance control following concussion using an accelerometer. J. Biomech..

[11] Matsushima A., Yoshida K., Genno H., Murata A., Matsuzawa S., Nakamura K., Nakamura A., Ikeda S. (2015). Clinical assessment of standing and gait in ataxic patients using a triaxial accelerometer. Cerebellum Ataxias.

[12] Senden R., Grimm B., Heyligers I.C., Savelberg H.H., Meijer K. (2009). Acceleration-based gait test for healthy subjects: Reliability and reference data. Gait Posture.

[13] Moe-Nilssen R., Helbostad J.L. (2004). Estimation of gait cycle characteristics by trunk accelerometry. J. Biomech..

[14] Zijlstra W., Hof A.L. (2003). Assessment of spatio-temporal gait parameters from trunk accelerations during human walking. Gait Posture.

[15] Ben Mansour K., Rezzoug N., Gorce P. (2015). Analysis of several methods and inertial sensors locations to assess gait parameters in able-bodied subjects. Gait Posture.

[16] Gonzalez R.C., Lopez A.M., Rodriguez-Uria J., Alvarez D., Alvarez J.C. (2010). Real-time gait event detection for normal subjects from lower trunk accelerations. Gait Posture.

[17] Maffiuletti N.A., Gorelick M., Kramers-de Quervain I., Bizzini M., Munzinger J.P., Tomasetti S., Stacoff A. (2008). Concurrent validity and intrasession reliability of the ideea accelerometry system for the quantification of spatiotemporal gait parameters. Gait Posture.

[18] Sekine M., Tamura T., Yoshida M., Suda Y., Kimura Y., Miyoshi H., Kijima Y., Higashi Y., Fujimoto T. (2013). A gait abnormality measure based on root mean square of trunk acceleration. J. Neuroeng. Rehabil..

[19] Sanchez M.C., Bussmann J., Janssen W., Horemans H., Chastin S., Heijenbrok M., Stam H. (2015). Accelerometric assessment of different dimensions of natural walking during the first year after stroke: Recovery of amount, distribution, quality and speed of walking. J. Rehabil. Med..

[20] Cicchetti D.V., Sparrow S.A. (1981). Developing criteria for establishing interrater reliability of specific items: Applications to assessment of adaptive behavior. Am. J. Ment. Defic..

[21] Haley S.M., Fragala-Pinkham M.A. (2006). Interpreting change scores of tests and measures used in physical therapy. Phys. Ther..

[22] Schreuders T.A., Roebroeck M.E., Goumans J., van Nieuwenhuijzen J.F., Stijnen T.H., Stam H.J. (2003). Measurement error in grip and pinch force measurements in patients with hand injuries. Phys. Ther..

[23] Lu W.S., Wang C.H., Lin J.H., Sheu C.F., Hsieh C.L. (2008). The minimal detectable change of the simplified stroke rehabilitation assessment of movement measure. J. Rehabil. Med..

[24] Huang S.L., Hsieh C.L., Wu R.M., Tai C.H., Lin C.H., Lu W.S. (2011). Minimal detectable change of the timed “up & go” test and the dynamic gait index in people with parkinson disease. Phys. Ther..

[25] Bland J.M., Altman D.G. (1999). Measuring agreement in method comparison studies. Stat. Methods Med. Res..

[26] Tura A., Raggi M., Rocchi L., Cutti A.G., Chiari L. (2010). Gait symmetry and regularity in transfemoral amputees assessed by trunk accelerations. J. Neuroeng. Rehabil..

[27] Sadeghi H., Allard P., Prince F., Labelle H. (2000). Symmetry and limb dominance in able-bodied gait: A review. Gait Posture.

[28] Kuan T.S., Tsou J.Y., Su F.C. (1999). Hemiplegic gait of stroke patients: The effect of using a cane. Arch. Phys. Med. Rehabil..

[29] Nilsson J., Thorstensson A. (1989). Ground reaction forces at different speeds of human walking and running. Acta Physiol. Scand..

[30] Demonceau M., Donneau A.F., Croisier J.L., Skawiniak E., Boutaayamou M., Maquet D., Garraux G. (2015). Contribution of a trunk accelerometer system to the characterization of gait in patients with mild-to-moderate parkinson’s disease. IEEE J. Biomed. Health Inform..

[31] Rispens S.M., Pijnappels M., van Schooten K.S., Beek P.J., Daffertshofer A., van Dieen J.H. (2014). Consistency of gait characteristics as determined from acceleration data collected at different trunk locations. Gait Posture.

